# Prognostic impact of phosphatase and tensin homolog status in recurrent IDH-wildtype glioblastoma receiving second-line therapy

**DOI:** 10.1093/noajnl/vdag196

**Published:** 2026-07-27

**Authors:** Eugenia Cella, Maurizio Polano, Marta Padovan, Elena Maria Marchesani, Alberto Bosio, Luca Bertero, Marta Maccari, Giulia Cerretti, Mario Caccese, Martina Corrà, Elisa Bennicelli, Matteo Lambertini, Sara Lonardi, Roberta Rudà, Giuseppe Lombardi

**Affiliations:** Department of Internal Medicine and Medical Specialties (DIMI), School of Medicine, University of Genova, Genova, Italy; Department of Oncology, San Paolo Hospital, Savona, Italy; Experimental and Clinical Pharmacology Unit, National Cancer Institute C.R.O.-IRCCS Aviano, Aviano, Italy; Department of Oncology, Oncology 1, Veneto Institute of Oncology IOV-IRCCS, Padova, Italy; Department of Neuroscience “Rita Levi Montalcini”, Division of Neuro-Oncology, University of Turin, Turin, Italy; Department of Oncology, Oncology 1, Veneto Institute of Oncology IOV-IRCCS, Padova, Italy; Department of Medical Sciences, Pathology Unit, University of Turin and Città della Salute e della Scienza University Hospital, Turin, Italy; Department of Oncology, Oncology 1, Veneto Institute of Oncology IOV-IRCCS, Padova, Italy; Department of Oncology, Oncology 1, Veneto Institute of Oncology IOV-IRCCS, Padova, Italy; Department of Oncology, Oncology 1, Veneto Institute of Oncology IOV-IRCCS, Padova, Italy; Department of Oncology, Oncology 1, Veneto Institute of Oncology IOV-IRCCS, Padova, Italy; Department of Oncology, Academic Oncology Unit, IRCCS Azienda Ospedaliera Metropolitana, Genova, Italy; Department of Internal Medicine and Medical Specialties (DIMI), School of Medicine, University of Genova, Genova, Italy; Academic Medical Oncology Unit, Ente Ospedaliero Ospedali Galliera, Genova, Italy; Department of Oncology, Oncology 1, Veneto Institute of Oncology IOV-IRCCS, Padova, Italy; Department of Neuroscience “Rita Levi Montalcini”, Division of Neuro-Oncology, University of Turin, Turin, Italy; Department of Oncology, Oncology 1, Veneto Institute of Oncology IOV-IRCCS, Padova, Italy

**Keywords:** biomarker, glioblastoma, PTEN, recurrence, regorafenib

## Abstract

**Background:**

Recurrent IDH-wildtype glioblastoma has a poor prognosis, and no validated molecular biomarker guides second-line treatment choices. Because phosphatase and tensin homolog (PTEN) alterations are frequent in glioblastoma and may influence therapy resistance, we assessed their association with outcomes in patients with recurrence of IDH-wildtype glioblastoma.

**Methods:**

This is a retrospective study including consecutive adults with first recurrence of IDH-wildtype glioblastoma treated between 2019 and 2022 with regorafenib, nitrosoureas, or bevacizumab. Phosphatase and tensin homolog alterations were assessed by next generation sequencing. Overall survival (OS) was analyzed using Kaplan-Meier estimates and multivariable Cox models adjusted for clinically relevant covariates. An exploratory weighted analysis compared regorafenib and nitrosoureas in PTEN wild-type tumors.

**Results:**

Among 226 patients, 128 (57%) had PTEN alterations. The prevalence was higher in patients who received regorafenib (66%) than in those treated with nitrosourea (48%) or bevacizumab (55%). In univariate analyses, PTEN alteration was associated with shorter survival with regorafenib (median OS, 9.4 vs 17.0 months; hazard ratio [HR], 2.04; *P* = .043) and nitrosoureas (6.9 vs 8.0 months; HR, 1.63; *P* = .027), but not with bevacizumab (HR, 1.23; *P* = .60). In multivariable analyses, PTEN alteration remained associated with worse survival with regorafenib (HR, 1.88; *P* = .016) and nitrosoureas (HR, 1.97; *P* = .020). In the overall cohort, PTEN alteration remained associated with worse survival (HR, 2.04; 95% confidence interval, 1.26-3.30; *P* = .0039), independently of O⁶-methylguanine-DNA methyltransferase promoter methylation.

**Conclusions:**

Phosphatase and tensin homolog alteration is a clinically relevant prognostic biomarker in recurrent IDH-wildtype glioblastoma and warrants prospective validation as a stratification factor.

Key Points

- Phosphatase and tensin homolog (PTEN) alterations were identified in more than half of molecularly profiled recurrent IDH-wildtype glioblastoma cases.
- PTEN alteration was associated with shorter overall survival in patients receiving second-line therapy.
- The adverse prognostic association of PTEN was observed across commonly used second-line treatment settings, but no significant PTEN-by-treatment interaction was identified.
- These findings support further evaluation of PTEN as a prognostic stratification factor at recurrence.

Importance of the Study

This multicenter real-world study supports the adverse prognostic association of phosphatase and tensin homolog (PTEN) alterations in recurrent IDH-wildtype glioblastoma across commonly used second-line treatment settings. Although no treatment-specific predictive effect was demonstrated, PTEN alterations may provide clinically relevant prognostic information for patient stratification at recurrence.

Glioblastoma (GBM) is the most common and aggressive primary malignant brain tumor in adults.^1^ Despite multimodal upfront treatment including maximal safe resection, radiotherapy, and temozolomide-based chemotherapy, prognosis remains poor, with a median overall survival (OS) of approximately 14-18 months from diagnosis.^2^ At disease recurrence, therapeutic options remain limited and include nitrosoureas, regorafenib, bevacizumab, and, in selected cases, temozolomide rechallenge or local therapies. In routine practice, treatment selection is still largely empiric because no validated molecular biomarker is available to guide second-line decision-making.^3^ Regorafenib showed encouraging activity in the phase II REGOMA trial,^4^ but this signal was not reproduced consistently across later datasets,^5^ reinforcing the impression that a subset of patients may derive benefit, whereas others do not. Nitrosoureas remain a widely used standard comparator despite modest efficacy.^5^^,^^6^ Bevacizumab has shown benefits in progression-free survival (PFS) and in controlling edema, but without improvement in OS.^7^^,^^8^

Together, these observations underscore the biological heterogeneity of recurrent glioblastoma and the need for clinically informative biomarkers.^9^ Phosphatase and tensin homolog (PTEN), located on chromosome 10q23.3, is a key tumor suppressor gene that negatively regulates the PI3K/AKT/mTOR signaling pathway and plays a central role in controlling cell growth, survival, and migration.^10^ Phosphatase and tensin homolog loss through mutation or deletion represents one of the most frequent molecular alterations in GBM and is considered an early event in gliomagenesis.^11-15^ Despite the high frequency of PTEN/PI3K pathway activation in gliomas, particularly in GBM, routine testing for these alterations is not currently recommended due to the lack of effective targeted therapies (ESCAT IIIA).^9^ Beyond glioma, PTEN loss has been associated with altered sensitivity to chemotherapy, radiotherapy, and targeted agents across multiple tumor types, suggesting a potential role in modulating treatment efficacy.^16-18^

The clinical significance of PTEN alterations in the setting of recurrent glioblastoma remains incompletely characterized. This is critical to assess also considering emerging evidence suggesting that PTEN alterations in glioblastoma are functionally heterogeneous and may shape treatment response through effects on tumor signaling and the immune microenvironment.^19^^,^^20^ We evaluated the prognostic significance of PTEN alterations in recurrent IDH-wildtype glioblastoma treated with standard second-line therapies, and examined its potential utility as a stratification factor across treatment groups.

## Methods

This is a retrospective, multicenter, real-world cohort study including consecutive adult patients with recurrent IDH-wildtype glioblastoma treated between 2019 and 2022 at the Veneto Institute of Oncology (Padua, Italy) and the University of Turin (Turin, Italy). Eligible patients were aged 18 years or older, had histologically confirmed IDH-wildtype glioblastoma according to the 2021 WHO classification, had received standard first-line treatment, and subsequently developed a radiologically documented first recurrence. Only patients who started systemic second-line therapy at first recurrence were included.

Second-line treatment consisted of regorafenib, nitrosoureas (lomustine or fotemustine), or bevacizumab, according to institutional practice, patient characteristics, drug availability, and multidisciplinary tumor board decision-making. Phosphatase and tensin homolog alteration was not used to guide treatment allocation.

Tumor molecular profiling was performed according to local availability using: FoundationOne CDx (Foundation Medicine) or Illumina TruSight Oncology 500 (TSO 500; Illumina). Phosphatase and tensin homolog and O⁶-methylguanine-DNA methyltransferase (MGMT) molecular analyses were performed on tumor tissue obtained at initial diagnosis rather than at recurrence, according to material available in routine clinical practice. No cross-platform harmonization of sequencing data was performed, and analyses were based on site-reported variant annotations.

Phosphatase and tensin homolog sequence alterations were derived from an annotated mutation file and reported according to standard Human Genome Variation Society (HGVS) nomenclature. Variant-level filtering retained PTEN alterations with protein-altering annotated consequences (including missense, nonsense, start/stop lost, and canonical splice-site variants) and variant allele frequency ≥5%, followed by protein-level normalization and aggregation at the patient level.

Phosphatase and tensin homolog status was dichotomized as altered vs wild type as the presence of at least 1 PTEN alteration meeting the above filtering criteria, or as a site-reported PTEN alteration in datasets without directly harmonizable variant-level records; all remaining cases were classified as PTEN wild type. Phosphatase and tensin homolog alterations were further characterized descriptively at the protein level. Where available, HGVS protein annotations were standardized and used to derive amino acid positions. Variants were mapped to canonical PTEN domains, including the phosphatase (DSPc), C2, and C-terminal regions, and summarized for descriptive and exploratory analyses. Systematic assessment of PTEN copy number alterations, chromosome 10q loss, loss of heterozygosity, and protein expression was not available for all cases. Therefore, PTEN wild-type classification in this study was based on the absence of detectable PTEN sequence alterations in the available molecular datasets and does not exclude alternative mechanisms of PTEN functional loss.

O⁶-methylguanine-DNA methyltransferase promoter methylation status was assessed on tumor tissue according to institutional routine practice using pyrosequencing or methylation-specific PCR. Tumors were classified as methylated, unmethylated, or indeterminate according to locally validated cutoffs.

The primary objective of the study was to assess the prognostic association between PTEN status and OS in patients with recurrent IDH-wild-type glioblastoma receiving second-line treatment.

### Statistical Analysis

Overall survival was defined as the time from second-line treatment initiation to death from any cause, with censoring at the date of last known follow-up for patients who were alive. Cox proportional hazards models were used to estimate HRs and 95% confidence intervals (CIs). For OS, a main-effects model including PTEN and treatment was fitted, followed by a model including a PTEN-by-treatment interaction term; models were compared using a likelihood ratio test. Cox proportional hazards regression models were used to estimate hazard ratios and 95% CIs. Multivariable models adjusted for age, Eastern Cooperative Oncology Group (ECOG) performance status, steroid use, repeat surgery at recurrence, and MGMT promoter methylation status.

To explore whether PTEN might have a treatment-specific effect, we fitted a comprehensive model including treatment group, PTEN status, and a PTEN-by-treatment interaction term.

Because treatment allocation was nonrandomized, an exploratory propensity score-based inverse probability of treatment weighting (IPTW) analysis using stabilized weights was performed in the subgroup of PTEN wild-type patients treated with regorafenib or nitrosoureas. The propensity score for receipt of regorafenib was estimated using baseline covariates measured before second-line treatment initiation, including age, sex, ECOG performance status, repeat surgery at recurrence, baseline steroid use, and MGMT promoter methylation status. Weighted Kaplan-Meier estimators and weighted Cox regression models with robust variance estimation were then fitted. Covariate balance before and after weighting was assessed using absolute standardized mean differences, with values below 0.10 considered indicative of adequate balance. Proportional hazard assumptions were evaluated using Schoenfeld residuals.

Given the limited sample size and observational design, the IPTW analysis was considered exploratory and was not intended to support causal inference or comparative treatment efficacy. All statistical tests were 2-sided, and *P* < .05 was considered statistically significant. Analyses were performed using R (version 4.5.2).

The study was conducted in accordance with the Declaration of Helsinki and approved by the local Ethics Committee (ANIMA study; IOV-2024-ANIMA). Written informed consent from surviving patients was obtained when required, or consent was waived according to local regulations for retrospective analyses.

## Results

A total of 226 patients with recurrent IDH-wildtype glioblastoma were included. At first recurrence, 96 patients received regorafenib, 92 received nitrosoureas, and 38 were treated with bevacizumab. Baseline characteristics differed across treatment groups (Table 1 and Figure S1). Patients receiving regorafenib were generally younger, more likely to have ECOG performance status 0-1 (94%), and more likely to have undergone repeat surgery (41%) at recurrence than those receiving nitrosoureas (22%) or bevacizumab (21%). In contrast, the bevacizumab cohort showed a less favorable clinical profile, including poorer performance status and higher baseline dexamethasone requirements, consistent with greater symptomatic burden at treatment initiation. O⁶-methylguanine-DNA methyltransferase promoter methylation status also differed across groups, with fewer indeterminate cases and a higher proportion of unmethylated tumors in the regorafenib cohort (53%).

**Table 1.** Baseline clinical and molecular characteristics according to second-line treatment

Patient characteristics	Overall	Nitrosoureas	Bevacizumab	Regorafenib	*P*-value^a^
Number of patients	226	92	38	96	<.001
Median age at treatment initiation, median (range), year	59 (21-82)	63 (36-82)	59 (33-76)	58 (21-76)	.03
Gender, *n* (%)					.157
Male	88 (39)	60 (65)	20 (53)	60 (63)	
Female	139 (61)	32 (35)	18 (47)	36 (38)	
ECOG PS, *n* (%)					<.001
0-1	181(81)	68 (74)	23 (66)	90 (94)	
≥2	42 (18)	24 (26)	12 (34)	6 (6)	
Unknown	3 (1)	0 (0)	3 (8)	0 (0)	
Steroid use					<.001
Median mg dexamethasone (range)	2 (0-12)	2 (0-8)	6 (4-8)	2 (0-8)	
MGMT, *n* (%)					.05
Unmethylated	98 (43)	36 (39)	11 (29)	51 (53)	
Methylated	88 (39)	38 (41)	16 (42)	34 (35)	
Missing/undetermined	40 (18)	18 (19)	11 (29)	11 (12)	
Resurgery *n* (%)					.012
Yes	67 (30)	20 (22)	8 (21)	39 (41)	
No	159 (70)	72 (78)	30 (79)	57 (59)	
PTEN status, *n* (%)					.046
Alterated	128 (57)	44 (48)	21 (55)	63 (66)	
Wild type	98 (43)	48 (52)	17 (45)	33 (34)	
PTEN domain, *n* (%)					.506
Phosphatase (DSPc)	50 (22)	20 (22)	10 (26)	20 (21)	
C2 domain	22 (10)	5 (5)	6 (16)	11 (11)	
Outside main domains	11 (5)	4 (4)	1 (3)	6 (6)	
Unmapped	143 (63)	63 (69)	21 (55)	59 (61)	

Abbreviations: ECOG PS, Eastern Cooperative Oncology Group Performance Status; GBM, glioblastoma; MGMT, O⁶-methylguanine-DNA methyltransferase; NGS, next generation sequencing; PTEN, phosphatase and tensin homolog.

a Kruskal-Wallis test for continuous variables; Fisher exact test for categorical variables.

### Overall Survival According to Second-Line Treatment

Median OS was 12 months (95% CI, 9.1-14) with regorafenib, 7.3 months (95% CI, 6.4-9.0) with nitrosoureas, and 8.6 months (95% CI, 6.8-12) with bevacizumab.

**Figure 1.**
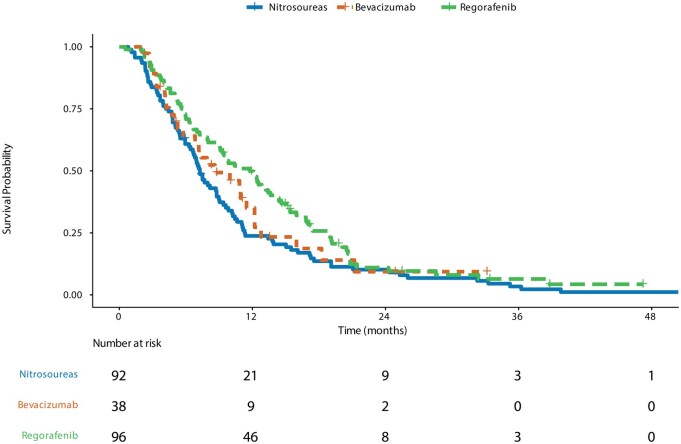
Kaplan-Meier overall survival according to second-line treatment group. Kaplan-Meier curves show overall survival from treatment initiation to death from any cause for patients treated with regorafenib, nitrosourea-based therapy, or bevacizumab. Censoring marks (+) indicate censored observations. The table below the plot displays the number of patients at risk at selected time points. Survival distributions were compared using the log-rank.

In the unadjusted analysis, patients treated with regorafenib showed longer OS compared with those receiving nitrosoureas (hazard ratio [HR] 0.69; 95% CI, 0.51-0.93; *P* = .015), however, treatment groups differed substantially in baseline clinical characteristics. No statistically significant difference was observed between bevacizumab and nitrosoureas (HR 0.85; 95% CI, 0.56-1.31; *P* = .461). The overall association between second-line treatment and OS was borderline significant at the global test level (*P* = .052) (Figure 1).

All univariable analyses are reported in Table S1. Importantly, several baseline prognostic factors, including age, MGMT promoter methylation, ECOG performance status, and second surgery, were associated with OS, and treatment allocation differed according to second surgery status.

### Distribution and Prognostic Impact of PTEN Alterations

Phosphatase and tensin homolog alterations were identified in 128 of 226 patients (57%). Their prevalence differed across treatment groups: 63 of 96 patients (66%) treated with regorafenib, 44 of 92 (48%) treated with nitrosoureas, and 21 of 38 (55%) treated with bevacizumab had PTEN-altered tumors. The distribution of individual PTEN sequence alterations is reported in Table S2, and the positional distribution across the PTEN protein is shown in Figure S2.

**Figure 2.**
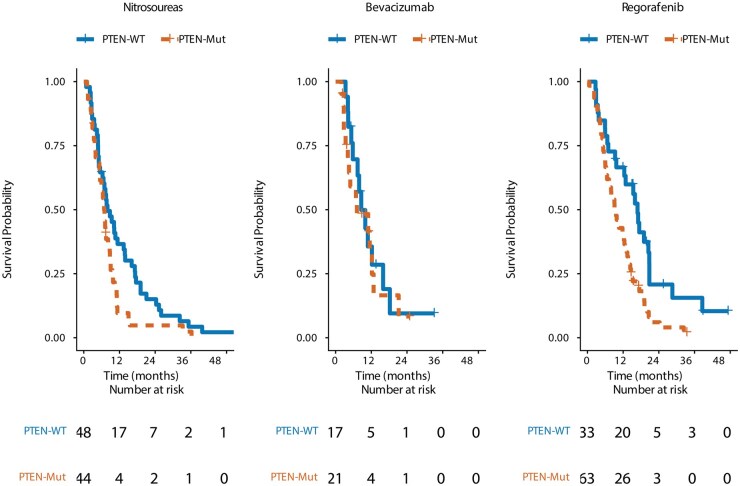
Kaplan-Meier stratified by PTEN alteration and second-line treatment. Abbreviation: PTEN, phosphatase and tensin homolog.

In univariate analyses, PTEN alteration was associated with shorter OS in patients treated with regorafenib (median OS, 9.4 vs 17.0 months; HR, 2.04; *P* = .043) and nitrosoureas (median OS, 6.9 vs 8.0 months; HR, 1.63; *P* = .027). No significant association between PTEN status and survival was observed in the bevacizumab cohort (HR, 1.23; *P* = .60). When restricting the analysis to patients treated with regorafenib or nitrosoureas (*n* = 188), PTEN alteration remained significantly associated with worse OS (HR, 1.66; 95% CI, 1.21-2.27; *P* = .001). Median OS estimates according to PTEN status are summarized in Table 2, and Kaplan-Meier curves within treatment groups are shown in Figure 2.

**Table 2.** Overall survival according to PTEN alteration within each treatment group

Treatment group	PTEN status	Median OS, months (95% CI)	HR for altered vs wild type (95% CI); *P*-value
Regorafenib	Wild type Altered	16.8 (12.4-20.8) 9.4 (7.2-12.5)	Reference 2.04 (1.26-3.30); .004
Nitrosureas	Wild type Altered	8.0 (6.6-13.8) 6.9 (5.5-8.8)	Reference 1.63 (1.06-2.51); .027
Bevacizumab	Wild type Altered	8.6 (7.4-NR) 7.2 (4.5-NR)	Reference 1.18 (0.56-2.49); 0.666

Hazard ratios were estimated using univariable Cox regression models within each treatment group.

Abbreviations: CI, confidence interval; HR, hazard ratio; NR, not reached; OS, overall survival; PTEN, phosphatase and tensin homolog.

In a comprehensive multivariable model including all second-line treatments (Table S3), PTEN alteration was independently associated with worse OS (HR, 1.63; 95% CI, 1.22-2.19; *P* = s.001), whereas no statistically significant interaction between PTEN alteration and treatment type was observed (*P* = .82 for interaction). These findings support a prognostic rather than treatment-predictive role for PTEN in this setting.

**Figure 3.**
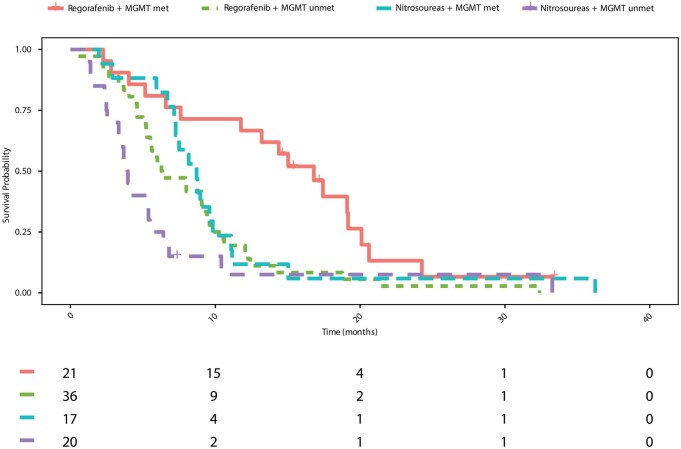
Adjusted overall survival by treatment (nitrosoureas vs regorafenib) and MGMT promoter methylation status Abbreviation: MGMT, O⁶-methylguanine-DNA methyltransferase.

O⁶-methylguanine-DNA methyltransferase promoter methylation retained a strong favorable prognostic effect in the overall model (HR, 0.55; *P* < .001) (Figure 3 and Table S3).

### Exploratory IPTW Analysis in PTEN Wild-Type Tumors

**Figure 4.**
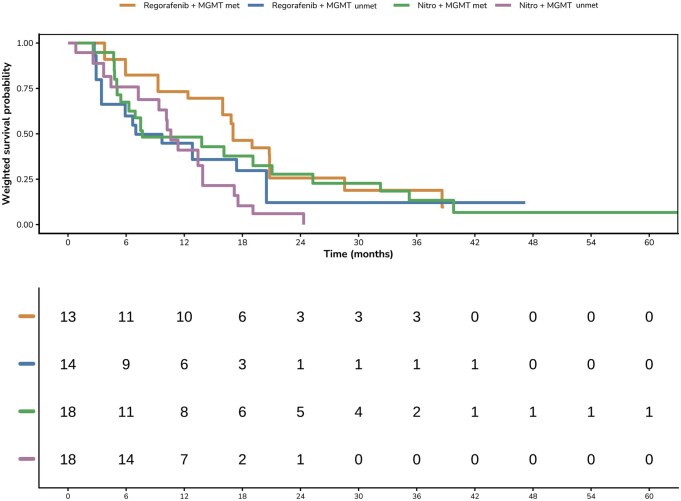
IPTW-weighted treatment and PTEN effects on overall survival. Forest plot showing weighted hazard ratios from Cox models after inverse probability of treatment weighting for the comparison between regorafenib and nitrosoureas. The upper panel shows the treatment effect within PTEN strata, and the lower panel shows the prognostic effect of PTEN alteration within treatment groups. PTEN alteration was associated with worse survival in both treatment groups, whereas no significant treatment-by-PTEN interaction was observed (*P* = .784 for interaction). Abbreviation: PTEN, phosphatase and tensin homolog.

Among PTEN wild-type patients treated with regorafenib or nitrosoureas, baseline imbalances were observed in age, ECOG performance status, repeat surgery at recurrence, steroid use, and MGMT promoter methylation status. After application of stabilized IPTW, covariate balance improved across measured baseline variables, as shown in Figure S3 and Table S4. In response to baseline imbalances between treatment groups, an exploratory stabilized IPTW analysis was performed in the PTEN wild-type subgroup treated with regorafenib or nitrosoureas. After weighting, all measured baseline covariates achieved adequate balance, with absolute standardized mean differences below 0.10 (Table S4 and Figure S3). In the IPTW-weighted Cox model with robust variance estimation, no statistically significant difference in OS was observed between regorafenib and nitrosoureas (HR, 0.76; 95% CI, 0.35-1.68; *P* = .50). O⁶-methylguanine-DNA methyltransferase promoter methylation was associated with a borderline favorable survival trend (HR, 0.55; 95% CI, 0.28-1.09; *P* = .087), while no significant treatment-by-MGMT interaction was detected. Inverse probability of treatment weighting-weighted OS curves and data comparing regorafenib and nitrosoureas in PTEN wild-type tumors are shown in Figure 4 and Table S4.

## Discussion

In this retrospective 2-center real-world study, PTEN alteration was independently associated with poorer survival in recurrent IDH-wildtype glioblastoma, with the clearest signal observed in patients treated with regorafenib and nitrosoureas. No significant association was observed in the bevacizumab subgroup, although this analysis was limited by sample size. Overall, our data indicate that PTEN alterations may help stratify prognosis at recurrence and warrant prospective evaluation within biomarker-informed treatment frameworks.

There are potential biological explanations for these findings. Phosphatase and tensin homolog loss results in constitutive activation of the PI3K/AKT/mTOR pathway and contributes to proliferation, invasion, and therapy resistance.^21-23^ In our cohort, PTEN alterations were associated with poorer outcomes among patients treated with nitrosoureas and regorafenib, suggesting that PTEN alteration identifies tumors with poorer clinical outcomes in these settings, although no treatment-specific predictive effect was demonstrated. In the setting of regorafenib, PTEN-deficient tumors may be less dependent on upstream angiogenic and oncogenic kinase signaling, thereby attenuating the efficacy of multikinase inhibition. Regorafenib exerts its antitumor activity through inhibition of angiogenic, stromal, and oncogenic kinases, including VEGFR, RAF, and KIT.^24^ Constitutive activation of downstream signaling pathways driven by PTEN loss may reduce tumor dependence on these upstream targets, thereby attenuating sensitivity to multikinase inhibition. In the setting of nitrosoureas, PTEN loss may promote resistance to DNA-damaging agents through impaired apoptosis and altered DNA damage response; these biological mechanisms may partially explain the inferior outcomes observed in this subgroup.^25^ Although mechanistic inference remains speculative in a retrospective clinical study, the consistency of the adverse association across adjusted models supports the biological relevance of PTEN status. In contrast, no significant association between PTEN alteration and survival outcomes was observed in patients treated with bevacizumab. The absence of a clear PTEN-associated signal in bevacizumab-treated patients may reflect a different therapeutic mechanism, limited statistical power, or both. Bevacizumab primarily acts through vascular normalization and edema control rather than direct tumor-cell cytotoxicity, which may partly explain why PTEN-driven tumor-intrinsic signaling had less obvious clinical impact in this subgroup. However, the relatively small number of bevacizumab-treated patients precludes firm conclusions.

Importantly, the prognostic impact of PTEN appeared to be independent of MGMT promoter methylation status, a well-established biomarker in glioblastoma, suggesting that PTEN may provide complementary biological information for patient stratification in the recurrent setting, rather than overlapping with MGMT-related mechanisms of treatment sensitivity.

Phosphatase and tensin homolog alterations are common in glioblastoma and biologically support a more aggressive phenotype through activation of the PI3K/AKT/mTOR pathway; however, their pure prognostic role has been inconsistent across studies. While meta-analytic data in glioma suggest that PTEN mutations are generally associated with shorter OS, results in glioblastoma have been less uniform, likely because of differences in assay methodology, molecular background, and treatment context.^26^ A translational analysis of the EORTC 26101^27^ trial where patients with recurrent glioblastoma were randomized to be treated with lomustina or lomustina plus bevacizumab showed that PTEN mutations were associated with shorter OS, supporting a prognostic adverse role of PTEN in progressive disease. Conversely, PTEN did not identify patients deriving a specific benefit from bevacizumab, indicating that PTEN should be regarded as a prognostic rather than a predictive biomarker in this setting. Therefore, our findings do not identify PTEN as a novel biomarker, but rather extend existing evidence within a real-world multicenter cohort including multiple commonly used second-line treatment strategies. Overall, these data together with our study support the interpretation of PTEN as a marker of unfavorable biology in recurrent glioblastoma.

Several limitations should be acknowledged. First, the retrospective design and nonrandomized treatment allocation introduce the potential for confounding by indication. Although multivariable adjustment and exploratory propensity score-based analyses were applied, residual confounding cannot be excluded. Second, molecular analyses were performed using different sequencing platforms, and PTEN alterations were grouped irrespective of mutation type or allelic status. In addition, systematic assessment of PTEN copy number alterations, chromosome 10q loss, loss of heterozygosity, homozygous deletion, and PTEN protein expression was not uniformly available. Consequently, some tumors classified as PTEN wild type may have harbored alternative mechanisms of PTEN inactivation. The heterogeneity and limited number of individual PTEN variants also precluded robust subtype-specific analyses. Third, matched germline DNA was not uniformly available because molecular profiling was performed as part of routine clinical practice. Therefore, a germline origin of PTEN alterations cannot be formally excluded, although this is considered uncommon in sporadic adult IDH-wildtype glioblastoma. Fourth, molecular analyses were performed on tissue obtained at initial diagnosis rather than at recurrence, preventing assessment of temporal molecular evolution and treatment-driven clonal selection. Yet, additional clinically relevant variables, including time from diagnosis to first recurrence and radiological tumor burden at recurrence, were not uniformly available and therefore could not be incorporated into adjusted analyses. Finally, the bevacizumab subgroup was relatively small, and the exploratory IPTW analysis was restricted to selected treatment groups. Therefore, these findings should be considered hypothesis-generating and require prospective validation.

Despite these limitations, our study has several strengths: a consecutive multicenter real-world population, molecularly characterized tumors, clinically meaningful second-line treatment groups, and a statistical framework explicitly distinguishing prognostic association from predictive interpretation. In a disease setting where treatment selection remains largely empiric, these data support PTEN as a promising stratification marker.

## Conclusion

In this retrospective 2-center real-world study, PTEN alteration emerged as a clinically relevant prognostic biomarker in recurrent IDH-wildtype glioblastoma. Phosphatase and tensin homolog alteration was consistently associated with poorer survival, supporting its potential value for prognostic stratification at recurrence rather than for treatment selection. Prospective validation is warranted to confirm the prognostic role of PTEN in recurrent glioblastoma.

## Supplementary Material

vdag196_Supplementary_Data

## Data Availability

The datasets generated and/or analyzed during the current study are available from the corresponding author on reasonable request.
