# Supplementary material for: Prognostic impact of phosphatase and tensin homolog status in recurrent IDH-wildtype glioblastoma receiving second-line therapy
Source: Neurooncol Adv. 2026 Jul 27;8(1):vdag196. doi: 10.1093/noajnl/vdag196 (PMC13484515; doi:10.1093/noajnl/vdag196)
Supplement: Supplementary file 1 [file vdag196_supplementary_data.docx]

# Supplementary Materials

| **Characteristic** | **N** | **Event N** | **HR** | **95% CI** | **p-value** |
| --- | --- | --- | --- | --- | --- |
| **Age treatment**  <60  > = 60 | 226 | 202 | —  1.36 | —  1.03, 1.80 | 0.031 |
| **Gender**  Female  Male | 226 | 202 | —  1.11 | —  0.83, 1.47 | 0.493 |
| **MGMT promoter methylation**  Unmethylated  Methylated | 186 | 166 | —  0.56 | —  0.41, 0.76 | <0.001 |
| **ECOG PS at start of second line**  1  2 | 223 | 199 | —  1.92 | —  1.35, 2.73 | <0.001 |
| **Second surgery**  No  Yes | 226 | 202 | —  0.69 | —  0.51, 0.94 | 0.018 |
| **PTEN**  Mut  WT | 226 | 202 | —  0.64 | —  0.48, 0.85 | 0.002 |
| **Second-line treatment**  Nitrosureas  Bevacizumab  Regorafenib | 226 | 202 | —  0.85  0.69 | —  0.56, 1.31  0.51, 0.93 | 0.461  0.015 |
| Abbreviation: CI = Confidence Interval, HR = Hazard Ratio | | | | | |

**Supplementary Table S1**. Univariable analysis of prognostic factors for overall survival

| **Characteristic** | **N = 226**^1^ |
| --- | --- |
| PTEN alteration |  |
| 253+1G>T | 1 (1.2%) |
| 3;splice site 209+1G>A | 1 (1.2%) |
| 634+2T>C; 253+5G>A | 1 (1.2%) |
| A39V | 1 (1.2%) |
| C105Y | 1 (1.2%) |
| C211fs*32 | 1 (1.2%) |
| D215fs*6 | 1 (1.2%) |
| D24fs*19 | 1 (1.2%) |
| E114* | 1 (1.2%) |
| E242fs*11 | 1 (1.2%) |
| E73fs*27 | 1 (1.2%) |
| G129R | 1 (1.2%) |
| G132D | 1 (1.2%) |
| G132S; R130* | 1 (1.2%) |
| G36 | 1 (1.2%) |
| H118L | 1 (1.2%) |
| H61L | 1 (1.2%) |
| H93R | 1 (1.2%) |
| I135K | 1 (1.2%) |
| I303fs*8 | 1 (1.2%) |
| K125E | 1 (1.2%) |
| K144del | 1 (1.2%) |
| K183fs*15 | 1 (1.2%) |
| K267fs30 | 1 (1.2%) |
| l101fs*6 | 1 (1.2%) |
| L193P | 1 (1.2%) |
| M134I | 1 (1.2%) |
| N276K; N329fs*14 | 1 (1.2%) |
| N329fs*14 | 1 (1.2%) |
| na | 1 (1.2%) |
| p.A151D | 1 (1.2%) |
| p.D52G | 1 (1.2%) |
| p.D92H | 1 (1.2%) |
| p.F90S | 1 (1.2%) |
| p.G251S | 1 (1.2%) |
| p.H141N | 1 (1.2%) |
| p.I168T | 1 (1.2%) |
| p.I33V | 1 (1.2%) |
| p.L325R | 1 (1.2%) |
| p.M134I | 1 (1.2%) |
| p.P190L | 1 (1.2%) |
| p.P95L | 1 (1.2%) |
| p.P96L | 1 (1.2%) |
| p.Q171X | 1 (1.2%) |
| p.Q17E | 1 (1.2%) |
| p.Q17X | 1 (1.2%) |
| p.R130X | 2 (2.4%) |
| p.R15S | 1 (1.2%) |
| p.R173C | 1 (1.2%) |
| p.T167A | 1 (1.2%) |
| p.T366I | 1 (1.2%) |
| p.T398S | 1 (1.2%) |
| p.X404C | 1 (1.2%) |
| p.Y155C | 1 (1.2%) |
| p.Y16X | 1 (1.2%) |
| p.Y68H | 1 (1.2%) |
| P190_F195>L | 1 (1.2%) |
| P248fs*5 | 1 (1.2%) |
| R130* | 1 (1.2%) |
| R173H | 1 (1.2%) |
| R335fs*9 | 1 (1.2%) |
| R378fs*37 | 1 (1.2%) |
| R41fs*3 | 1 (1.2%) |
| S170I | 1 (1.2%) |
| S170N | 1 (1.2%) |
| SPLIC SITE 635-1g>A | 1 (1.2%) |
| splice site 1027-2A>G | 1 (1.2%) |
| splice site 109+2 delT | 2 (2.4%) |
| splice site 209+1_209+2 delGT | 1 (1.2%) |
| splice site 209+1_209+2delGT | 1 (1.2%) |
| splice site 253+1G>A | 1 (1.2%) |
| splice site 253+5G>T | 1 (1.2%) |
| splice site 635-2A>T | 1 (1.2%) |
| splice site 80-1_89 del GATATTTATCC | 1 (1.2%) |
| T319fs*1 | 3 (3.6%) |
| V54fs*45 | 1 (1.2%) |
| V85fs*14 | 1 (1.2%) |
| W274 | 1 (1.2%) |
| W274G | 1 (1.2%) |
| Y76fs*1 | 1 (1.2%) |
| Unknown | 142 |
| ^1^n (%) | |

**Supplementary Table S2.** PTEN status was summarized as a categorical variable reflecting wild-type PTEN, functionally altered PTEN, or variants of unknown significance. The distribution of individual PTEN sequence alterations is reported

**Supplementary Figure S1.** Alluvial diagram illustrating the allocation pathway of second-line treatment according to baseline patient characteristics (sex, ECOG performance status, MGMT promoter methylation status, and age)

**Supplementary Figure S2.** Lollipop plot showing the distribution and frequency of PTEN sequence alterations across the protein. Each lollipop represents a unique amino acid position; height indicates the number of samples. Colors denote mutation type. Functional domains are shown below.

| **Characteristic** | **HR** | **95% CI** | **p-value** |
| --- | --- | --- | --- |
| **PTEN status**  WT  Mut | —  1.63 | —  1.22, 2.19 | 0.001 |
| **Treatment**  Nitrosureas  Bevacizumab  Regorafenib | —  0.73  0.68 | —  0.46, 1.14  0.50, 0.94 | 0.2  0.019 |
| **Age treatment** | 1.01 | 0.99, 1.02 | 0.3 |
| **MGMT**  Methylated  Missing/indeterminate  Unmethylated | —  1.64  2.09 | —  1.09, 2.48  1.51, 2.89 | 0.018  <0.001 |
| **ECOG PS**  ≥2  0-1 | —  0.60 | —  0.41, 0.88 | 0.009 |
| **Second surgery**  No  Yes | —  0.70 | —  0.51, 0.96 | 0.025 |
| **Gender**  Female  Male | —  1.10 | —  0.82, 1.49 | 0.5 |
| Abbreviations: CI = Confidence Interval, HR = Hazard Ratio | | | |

**Supplementary Table S3**: Cox multivariate complete data

**Supplementary Figure S3. Covariate Balance Before and After Inverse Probability of Treatment Weighting.**
Absolute standardized mean differences for baseline covariates are shown before (unadjusted) and after inverse probability of treatment weighting. The dashed vertical line indicates the prespecified balance threshold of 0.1. After weighting, all covariates achieved adequate balance

*: indicates variables for which the displayed value is the raw (unstandardized) difference in means.

| **Variable** | **Nitrosoureas** | **Regorafenib** | **SMD before IPTW** | **SMD after IPTW** |
| --- | --- | --- | --- | --- |
| Age at second-line treatment, years | 60 (12) | 54 (14) | 0.500 | 0.051 |
| Sex |  |  | 0.189 | 0.041 |
| Female | 10 (30.3%) | 11 (39.3%) |  |  |
| Male | 23 (69.7%) | 17 (60.7%) |  |  |
| ECOG performance status |  |  | 0.287 | 0.017 |
| 0-1 | 26 (78.8%) | 25 (89.3%) |  |  |
| ≥2 | 7 (21.2%) | 3 (10.7%) |  |  |
| Repeat surgery at recurrence |  |  | 0.323 | 0.030 |
| No | 25 (75.8%) | 17 (60.7%) |  |  |
| Yes | 8 (24.2%) | 11 (39.3%) |  |  |
| Baseline steroid use |  |  | 0.076 | 0.037 |
| No | 13 (39.4%) | 10 (35.7%) |  |  |
| Yes | 20 (60.6%) | 18 (64.3%) |  |  |
| MGMT promoter methylation |  |  | 0.162 | 0.040 |
| Unmethylated | 15 (45.5%) | 15 (53.6%) |  |  |
| Methylated | 18 (54.5%) | 13 (46.4%) |  |  |
| **Supplementary Table S4**. Covariate balance before and after stabilized IPTW in PTEN wild-type patients treated with regorafenib or nitrosoureas.  Values are mean (SD) for age and n (%) for categorical variables; SMD = standardized mean difference; IPTW = inverse probability of treatment weighting; absolute SMD values are reported. Values <0.10 were considered indicative of adequate covariate balance. Stabilized inverse probability weights were estimated from a propensity score model including age, sex, ECOG performance status, repeat surgery at recurrence, baseline steroid use, and MGMT promoter methylation status. | | | | |
|  | | | | |
|  | | | | |
